# ENTPRISE-X: Predicting disease-associated frameshift and nonsense mutations

**DOI:** 10.1371/journal.pone.0196849

**Published:** 2018-05-03

**Authors:** Hongyi Zhou, Mu Gao, Jeffrey Skolnick

**Affiliations:** Center for the Study of Systems Biology, School of Biological Sciences, Georgia Institute of Technology, Atlanta, Georgia, United States of America; UMR-S1134, INSERM, Université Paris Diderot, INTS, FRANCE

## Abstract

To exploit the plethora of information provided by Next Generation Sequencing, the identification of the genetic mutations responsible for disease in general or cancer in particular, among the thousands of neutral germline or somatic variations is a crucial task. Genome-wide association studies for the detection of disease-associated genes or cancer drivers can only identify common variations or driver genes in a cohort of patients. Thus, they cannot discover unique disease-associated mutations or cancer driver genes on a personal basis. Moreover, even when there are such common variations, their significance is unknown. Here, we extend the machine learning based approach ENTPRISE developed for predicting the disease association of missense mutations to frameshift and nonsense mutations. The new approach, ENTPRISE-X, is shown to outperform the state-of-the-art methods VEST-indel and DDIG-in for predicting the disease association of germline frameshift mutations in terms of balanced measure Matthew’s correlation coefficient, MCC, with a MCC of 0.586 for ENTPRISE-X, versus 0.412 by VEST-indel and 0.321 by DDIG-in, respectively. Large scale testing on the ExAC dataset shows ENTPRISE-X has a much lower fraction of 16% of variations classified as disease causing, as compared to VEST-indel’s 26% and DDIG-in’s 65% of predictions as being disease-associated. A web server for ENTPRISE-X is freely available for academic users at http://cssb2.biology.gatech.edu/entprise-x.

## Introduction

Identifying the causative genetic variations of a disease or cancer is a key step towards diagnosis and cure. A typical patient has thousands of genetic variations involving thousands of genes. It is thus a nontrivial task to pinpoint the single variation or group of variations responsible for the given disease. Traditionally, genome wide association studies (GWAS) are employed for detecting common variations among a cohort of same disease patients [1, 2]. Due to the heterogeneity of human disease [3], GWAS can only cover a small fraction of patients. Thus, for the majority of patients, new approaches for personalized detection of disease-associated variations are needed. Since disease is a complex system phenomenon that is often caused by the synergetic effects of many factors including genetic, environmental, and life style effects, one can only infer that a specific mutation is associated with a disease, which means it could contribute to the onset of a disease. Even so, it might not be the only cause of the disease. A mutation, in particular frameshift and nonsense ones, could result in a loss or gain of function of the protein. If the function of the normal protein is essential for life, the mutated protein with functional consequences could result in disease subject to extrinsic factors. Because of their importance, many methods have been developed to predict disease-associated mutations [4–12]. Some can also help annotate somatic mutations in cancer [13]. Approaches specific for cancer driver mutations have also been developed [14–17]. More generally, our recently developed machine learning based method ENTPRISE outperforms previous methods in terms of the ability to distinguish neutral from disease-associated missense mutations in the same protein, false positive rate, and performance in cancer driver prediction [18].

Although ENTPRISE has outstanding performance for both Mendelian and cancer driver mutations and requires only single patient exome information, like many other methods, it can only handle missense amino acid substitutions. In practice, based on the v76 version of the COSMIC database [19], while 64.4% of the mutations are missense amino acid substitutions, 21.7% are silent, 8.4% are nonsense and frameshift mutations, 1.4% are in-frame indels (insertions/deletions), and the remaining 4.1% are uncharacterized. Furthermore, since both nonsense and frameshift mutations disrupt protein structure more severely than missense mutations and in-frame indels, they are more likely to have pathogenic consequences. In that regard, there are a number of methods dedicated to assessing the disease association of frameshift and/or nonsense mutations [17, 20–22]. However, as in the case of missense variations, these approaches often have quite high false positive rates, which limit their practical application when applied to entire exomes.

In this work, we extend the ENTPRISE approach [18] that successfully classifies the disease association of missense mutations to include nonsense and frameshift mutations. Since both nonsense and frameshift mutations disrupt protein structure at a given location within a protein’s sequence, we will treat them identically. ENTPRISE uses a set of features derived from the protein’s sequence and predicted tertiary structure in a boosted tree regression method [23] to learn a model from the training dataset. Here, we shall use similar features derived from predicted protein structures and include additional features related to the global functionality of the protein. The extended approach which is called ENTPRISE-X will be benchmarked against the best current state-of-the-art DDIG-in [20] and VEST-indel [21] methods for frameshift and nonsense mutations. DDIG-in is a SVM-based machine-learning method [24] that uses both DNA and protein sequence information as well as predicted accessible surface area and can address both frameshift and nonsense variations. VEST-indel deals with frameshift and in-frame variations and uses Random Forest classifiers [25] to train a model using 24 features including a “PubMed” feature for disease relevance. In addition to comparing to these established approaches, we also perform a large scale test on the ExAC dataset of 60,706 exomes from unrelated individuals [3]. While ExAC is not necessarily completely neutral (i.e. all variations are not disease associated), the number of variations classified by a method to be disease-associated should be strongly correlated with the false positive rate of the method. The method developed in this work is useful for personalized Mendelian disease and protein target identification and is freely available for academic users at http://cssb2.biology.gatech.edu/entprise-x.

## Materials and method

### Datasets

#### Training set

Pathogenic data (ClinVar) were downloaded from the NCBI site ftp://ftp.ncbi.nlm.nih.gov/pub/clinvar/tab_delimited/. Two neutral data sets are used. One is EPS6500 from http://evs.gs.washington.edu/EVS/ [26], and the other is from the 1000 Genomes Project phase 3 data [27]. The mutations employed for training exclude mutations in proteins having sequence identity ≥ 35% to any proteins in the test data set described below.

#### Testing set

To compare ENTPRISE with VEST-indel and DDIG-in, we use the same frameshift testing set as employed in the VEST-indel [21]. It consists of 184 pathogenic mutations from the ClinVar database and 1,340 neutral ones from the SIFT-indel method constructed from inter-species multiple sequence alignment [22]. After mapping the protein sequences to structures in our predicted human exome protein structure database [18], we obtain 82 pathogenic and 1,025 neutral frameshift mutations. Since there are no independent data for comparing methods on nonsense mutations, we adopt a similar 10 fold cross-validation approach as was employed in the DDIG-in method [20] to benchmark ENTPRISE-X for nonsense mutations.

#### ExAC set

For large scale testing of our method in comparison to existing algorithms, we downloaded the ExAC dataset [28] that excludes the Cancer Genome Atlas (TCGA) data (http://cancergenome.nih.gov/). The goal of ExAC is to provide a global “reference set” for filtering out harmless genetic variants observed in patients with some disease. However, since the data only excluded childhood diseases, it could still contain variations causing disease in adults. The ExAC variations excluding the Cancer Genome Atlas (TCGA) data are considered to be mostly, but not completely, neutral [28]. A method with a larger false positive rate will result in a larger fraction of data as being classified as disease causing. Thus, to a rough first approximation, this large-scale test can be used to estimate the false positive rate of a method. After mapping proteins to our predicted protein structure database and excluding mutations in proteins having a sequence identity ≥ 35% to any protein in the training set, we obtained 56,917 putative neutral frameshift and 45,131 putative neutral nonsense mutations.

A brief summary of training and testing datasets on frameshift and nonsense used for ENTPRISE-X mutations are given in Table 1.

**Table 1:** Summary of variations in the ENTPRISE-X training and testing data sets.

**Data Set**				**Usage**
**Training set**				1. For training a model in future applications. 2. For feature reduction. 3. For large scale, ten-fold cross-validation test on nonsense mutations in comparison to DDIG-in, and on frameshift mutations in comparison to DDIG-in & SIFT-indel (see Table 3).
**Frameshift**		**Nonsense**		
Pathogenic	Neutral	Pathogenic	Neutral	
ClinVar: 6,513	ESP6500: 1,604	ClinVar: 5,023	ESP6500: 181	
	1000 GP: 366		1000 GP: 3,171	
Total numbers (sum of each column)				
6,513	1,970	5,023	32,51	
**Independent testing sets (not used in training)**				**Usage**
**VEST-indel set**				For test on frameshift variations in comparison to VEST-indel & DDIG-in methods (see Table 2).
**Frameshift**		**Nonsense**		
Pathogenic	Neutral	Pathogenic	Neutral	
ClinVar: 82	Inter-species: 1,025	─	─	
ExAC set				For large scale false positive rate test on frameshift & nonsense variations in comparison to the VEST-indel & DDIG-in methods
─	ExAC: 56,917	─	ExAC: 45,131	

### Features and machine learning

Owing to the success of the features in the ENTPRISE method [18] for missense mutations, we tested the same features for frameshift and nonsense mutations. These are derived from the protein’s sequence and predicted protein three-dimensional (3D) structural information. However, since for nonsense mutations the mutated protein has no amino acids after the mutation, the features describing the amino acid types of the mutated protein in ENTPRISE are not needed for nonsense mutations, whereas the features describing the amino acid type before the mutation are kept. For frameshift mutations, since the major result is similar to nonsense mutations (dysfunction of the part after mutation point), we also neglect the amino acid type right after the mutation. A detailed description of these features is found in Ref. [18]. Here, we give a brief summary and describe the newly introduced features specific for frameshift and nonsense mutations:

(a) The reference amino acid type of the mutated protein position. This requires 20 variables to represent the 20 types of amino acids. For a given residue type, the specific variable is set to the value of 1 and the rest of the variables are set to 0. This feature reflects the fact that mutations of certain types of amino acids are more likely to cause disease than others [29]. The wild type amino acid as well as the following contact composition and entropy information encode the functional importance of the mutated position when the protein functions normally.
(b) 20 variables for the amino acid composition of the residues in contact with the mutated protein position. This reflects the protein’s local structural environment at the mutated position and is defined as the amino acid composition of all residues whose C_α_ atom is within 12 Å from the mutated position’s C_α_ atom assuming that the structure of the protein is in the same native conformation. The consideration of this feature is that if a mutation is at or close to a protein-protein interface or protein-ligand binding pocket, the mutation is more likely to be disease associated [29]. Since certain residues are usually conserved in interface and binding sites, the amino acid composition around the mutated position encodes information if the mutation is close to an interface or binding pocket. Since for the majority of human proteins, experimental structures are not available, this work uses predicted protein structures from TASSER^VMT^ [30] as in ENTPRISE [18]. The composition is calculated by
  q(a)=N(a)/ΣN(b), (1)
  where *N*(*a*) is the number of residues of type *a* contacting the mutated position, and ∑*N*(*b*) sums over all 20 types of residues.
(c) 20 variables for the composition of the domain that contains the mutated protein position. The reason for considering this feature is that certain domains/proteins are more likely to be disease associated than others [7]. Proteins/domains are distinguishable by their amino acid composition. This feature encodes the characteristics of the domain at the mutated position. The domains of the mutated proteins are predicted by threading the sequence using the fast HHpred threading algorithm [31] against the SCOP domain database [32] in which domains are manually defined. The computation of domain composition is the same as Eq (1) with *N*(*x*) being replaced by the number of residues of type *x* in the whole domain.
(d) Sequence entropy of the mutated protein position. This characterizes the evolutionary conservation at the mutated position. Our assumption is that evolutionarily conserved residues are either functionally and/or structurally important. The sequence entropy is given by
  $$S_{r}=S-<S>,$$ (2)
  where $S=-\sum_{l=1}^{20}f_{l}\text{ln}\left(f_{l}\right)$ is the sequence entropy of the given position, *f*_*l*_ is the normalized amino acid frequency profile generated by sequence search method PSI-BLAST [33]. <> stands for averaging over the entire protein sequence.
  The above 61 features are the same as in the ENTPRISE method and describe the features of the mutated position. We next introduce five additional new features that describe partial or whole protein functionality. The newly introduced features specific for frameshift & nonsense mutations are:
(e) Fraction of the affected protein structure defined as (*N*_*r*_ − *k*)/*N*_*r*_ where *N*_*r*_ is the total number of residues of the protein, k is the sequential position of the mutation from the N-terminus. This feature describes how much of the protein is affected by the mutation that disrupts the structure. Our assumption is that the greater the fraction of the protein that is affected, the more likely it is that the mutation is disease associated.
(f) Essentiality of mutated protein. If the protein is homologous (defined as having protein sequence identity > 35%) to an essential protein in the database of essential genes [34], a feature with a value of one is set; otherwise, it is set to zero. The consideration of this feature is based on the idea that if the protein is essential, its functional change is more likely to be associated with disease.
(g) Pathogenicity of the affected part of the protein. This feature is derived from the ENTPRISE calculation on the whole protein sequence. It reflects the average effects of the disrupted structure if all the positions have a missense mutation in turn, one position a time. The assumption is that if the probability of a missense mutation being disease associated in the missing dysfunctional part of a protein is larger, a frameshift or nonsense mutation that disrupts this region will be more likely to be disease associated. For any position in the reference protein sequence, there are 19 possible missense mutations. The pathogenicity of a position is defined as the ratio of the number of mutations having an ENTPRISE score ≥ 0.45 divided by 19. (Note that an ENTPRISE score ≥ 0.45 is considered to be pathogenic). The pathogenicity of the affected part of the protein is the average pathogenicity of all positions after the nonsense/frameshift position in the sequence.
(h) Disease involvement of the protein. This feature provides the information that a protein is associated with disease when its function is altered. It is obvious a consideration for a mutation on the protein to be disease associated or not when it is mutated. If the protein is related to any disease as defined by the GeneCards database [35], this feature receives a value of one. In addition, we have collected gene-disease associations from other two sources [36, 37] that were used in our recent work [38] for predicting gene-disease association. If a protein is associated with any disease in this additional dataset, this feature receives another value of one. In all other cases, this feature is set to zero.
(i) Number of protein-protein interactions inferred from the HIPPIE protein-protein interaction database [39]. This feature reflects how many other proteins that it might affect if the protein’s function changes because of the mutation. Its consideration for disease association is that a protein with more interactions will likely affect more biological processes when its function is altered, and thus this increases the possibility of a mutation being disease associated.
  We shall employ the machine learning boosted tree regression method that has been employed in many other applications [23, 40]. It generates a sequence of decision trees; each grows on the basis of the residuals of all previous trees [23, 41]. Here, a decision tree regression is implemented with a maximal depth of eight. The scoring function is represented as a boosted decision tree [23]:
  $$f(x)=\sum_{m=1}^{N_{tree}}\varepsilon T_{m}.$$ (3)
  Where *T*_*m*_ is a decision tree, *ε* is the shrinkage factor or learning rate, and *N*_*tree*_ is the number of trees. Two objective function values are adopted: 1 for disease-associated mutations, and 0 for neutral mutations. The learning rate *ε* is set to 0.005 and the number of trees, *N*_*tree*_, is set to 2000. These values are purely empirical and were not optimized. As mentioned above, we treat frameshift and nonsense mutations exactly the same. Since in the training sets, the total number of disease-associated mutations (11,536) is roughly twice the total number of neutral mutations (5,222), to train the model on balanced datasets, we shall train two models: one with a randomly selected half of the disease-associated mutations and all neutral ones, the other with the remaining half of the disease-associated mutations and all neutral ones. This way, both models are trained on balanced datasets. In prediction/testing, a prediction will be given by the average score from the two models. In case of a jackknife or cross-validation test, the same training/testing protocol was applied to the training/testing subsets.

### Feature reduction

Overall, we have tested the 66 features described above. However, not all types of features are useful. We shall employ a feature reduction procedure to eliminate features that have negative impact on performance. To this end, we cluster the whole training proteins into 10 clusters with a 35% protein sequence identity cutoff and employ a leave one cluster out cross validation (LOOCV) test (also see ten-fold cross-validation subsection). Then, for each cluster, we use nine clusters for training and the one that is left out for testing. To eliminate features, for each type of feature, we remove it from training and perform a LOOCV test; if the feature’s removal results in a better Matthew’s Correlation Coefficient (MCC) with a default cutoff of 0.5, then the feature is discarded. As a result of this procedure, the 20 features of domain composition (type (c) above) were eliminated, leaving us with total of 46 final features including all five newly introduced features.

## Results

### Comparison to other methods

**Fig 1:**
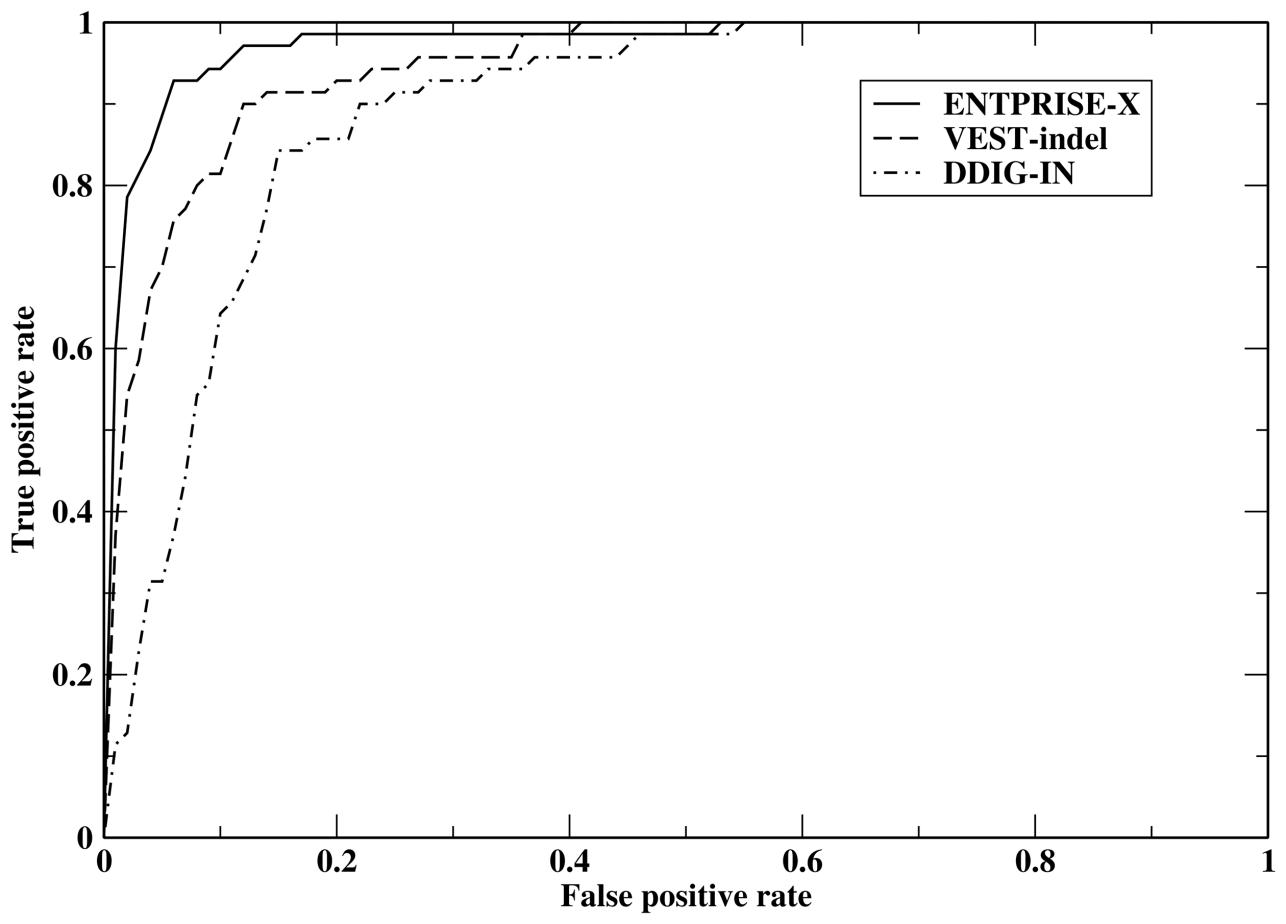
Receiver operating characteristic curves of ENTPRISE-X, VEST-indel and DDIG-in.

We next tested ENTPRISE-X using the 46 final features for frameshift mutations on the VEST-indel test set [21] and compared the results with the VEST-indel and DDIG-in methods [20, 21] which according to Ref [21] are the two most accurate methods for frameshift variations. The results are presented in Table 2. Comparison of different approaches is made on the consensus subset (all three methods have predictions) of the full test set. The full set has 82 pathogenic and 1025 neutral mutations, whereas the consensus has 70 pathogenic and 914 neutral mutations. The difference in numbers of cases is due to the fact that some methods like DDIG-in cannot interpret all of the input data. Results for VEST-indel (http://www.cravat.us/CRAVAT) and DDIG-in (http://sparks-lab.org/ddig/) are obtained from their respective webservers. Table 2 clearly shows that ENTPRISE-X with a default cutoff score 0.5 has the best overall performance in terms of its Matthew’s Correlation Coefficient and F-score defined as 2(precision×recall)/(precision+recall), where precision = (true positive)/(true positive + false positive), recall = (true positive)/(true positive + false negative). It also has the lowest false positive rate defined as (the number of neutral variations classified as disease-associated)/(the total number of neutral variations) and is related to specificity by 1-specificity. DDIG-in has the worst MCC and F-score. It tends to over-predict disease association and thus has the largest false positive rate. VEST-indel performs in the middle for all measures. In Fig 1, we compare the Receiver Operating Characteristic (ROC) of ENTPRISE-X, VEST-indel and DDIG-in. Fig 1 shows that ENTPRISE-X performs significantly better than VEST-indel and DDIG-in, especially in the low false positive regime that is the most important for practical application. For example, at a false positive rate of 2%, ENTPRISE-X has a true positive rate of 66%, whereas VEST-indel only has a 33% true positive rate.

**Table 2:** Performance on the VEST-indel test set for frameshift variations.

Method	MCC^b^	Sensitivity	Specificity	F-score^c^	False positive rate	False discovery rate
**Evaluation on the consensus subset**^a^						
ENTPRISE-X	0.626 (0.749)	0.943	0.916	0.620 (0.767)	8.4%	54%
VEST-indel	0.440 (0.585)	0.914	0.814	0.421 (0.615)	18.6%	73%
DDIG-in	0.321 (0.441)	0.943	0.663	0.297 (0.439)	33.7%	82%
**Evaluation on the full test set**						
ENTPRISE-X	0.586	0.878	0.912	0.590	8.8%	55%
Baseline^d^	0.323	0.988	0.621	0.294	37.9%	83%
Baseline^e^	0.224	0.598	0.775	0.271	22.5%	83%
ENTPRISE-X_1^f^	0.570	0.878	0.905	0.574	9.5%	57%
ENTPRISE-X_2^f^	0.555	0.854	0.905	0.562	9.5%	58%
ENTPRISE-X_10alt^f^	0.587±0.006	0.887±0.006	0.910±0.003	0.590±0.006	9.0%±0.3%	55.8%±0.7%
ENTPRISE-X-nolocal	0.481	0.707	0.914	0.509	8.6%	60%
ENTPRISE-X-nonew	0.099	0.793	0.390	0.168	61.0%	90%
ENTPRISE-X-noratio	0.513	0.890	0.871	0.509	12.9%	64%
ENTPRISE-X-noessential	0.574	0.890	0.903	0.575	9.7%	58%
ENTPRISE-X-nopathogen	0.543	0.866	0.896	0.546	10.4%	60%
ENTPRISE-X-nodisease	0.368	0.683	0.859	0.396	14.1%	72%
ENTPRISE-X-nointeract	0.586	0.890	0.909	0.588	9.1%	56%

^a^ To be fair to all methods, only the consensus mutations of three methods are evaluated in comparison to the other methods.

^b^ Matthew’s Correlation Coefficient. The numbers in parentheses are the maximal possible values.

^c^ 2(precision×recall)/(precision+recall), where precision = (true positive)/(true positive + false positive), recall = (true positive)/(true positive + false negative). Numbers in parentheses are the maximal possible values.

^d^ When only the feature representing if the gene is disease-associated or not is used.

^e^ When only the feature representing if the gene is essential or not is used.

^f^ When using one of the 2 models trained on each half of the pathogenic data and training ENTPRISE-X for 10 different random partitions of the pathogenic part of the training set were used.

Since we have used the feature representing whether the given gene is disease-associated or not as learned from literature (similar to the “PubMed” feature in VEST-indel [21]), it is of interest to establish how well it performs when this is the only feature used for prediction. We call this scenario the *baseline* method, with the results also given in Table 2. Clearly, it has a too large false positive rate and the overall performance is much worse than ENTPRISE-X. It is also interesting to use the essentiality of protein as another baseline approach. It has much lower sensitivity and specificity than those of ENTPRISE-X. We tested another version of ENTPRISE-X that removes all the features encoding local information of the mutated position ((a)-(d)) used in ENTPRISE. This scenario is called ENTPRISE-X-nolocal and is shown in Table 2. It too has worse sensitivity and overall performance than full ENTPRISE-X. It is also important to examine the significance of the five new features by removing them from the 46 features and the resulting variant is called “ENTPRISE-X-nonew”, its performance is much worse than that of ENTPRISE-X (see Table 2). This indicates that the newly introduced features provide the major contribution to performance of ENTPRISE-X. It is also interesting to see how different the individually trained models are from the default (average of two models) prediction. The results are shown in Table 2 with names ENTPRISE-X_1 and ENTPRISE-X_2. They differ slightly from each other and are slightly worse than the default prediction.

To see the importance of new features, we performed five separate tests by removing each of the five new features, one at a time:

ENTPRISE-X-noratio—removing the fraction of affected protein part;
ENTPRISE-X-noessential—removing the essentiality of protein;
ENTPRISE-X-nopathogen—removing the pathogenicity score predicted by ENTPRISE;
ENTPRISE-X-nodisease—removing the disease involvement of the protein;
ENTPRISE-X-nointeract—removing number of protein-protein interactions.

Table 2 shows that the order of the effect from strongest to weakest as ranked by their MCC & F-score are: (1) disease involvement of the protein; (2) fraction of the affected protein structure part; (3) pathogenicity of the affected part of the protein as predicted by ENTPRISE; (4) essentiality of mutated protein; and (5) number of protein-protein interactions. The effect of the number of protein-protein interactions has no visible effect on the MCC but has a small effect on the F-score and false positive rate.

**Fig 2:**
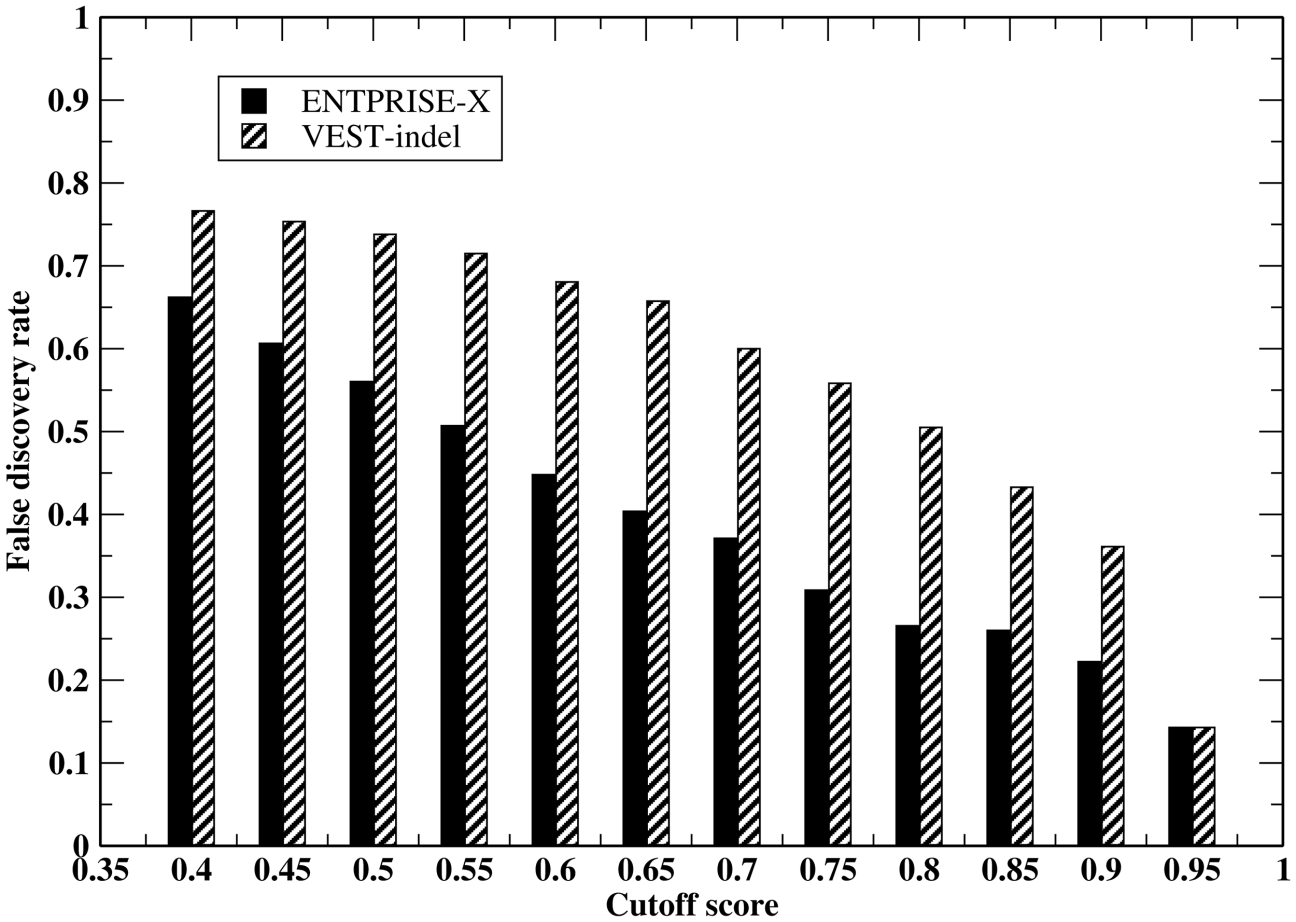
False discovery rate by the ENTPRISE-X and VEST-indel methods at various cutoffs on the VEST-indel test set.

In practice, due to the fact that neutral variations always dominate over true disease-associated variations, even a small false positive rate will result in large number of false positive predictions. We employ the “false discovery rate” (FDR) defined as (number of false positive predictions)/(total number of predictions). This rate depends not only on the false positive rate, but also on the relative abundance of true positives and true negatives in the evaluated data set. Fig 2 compares the false discovery rate by ENTPRISE-X and VEST-indel methods at various cutoffs on the VEST-indel test set. At the default cutoff score 0.5, both methods are dominated by false positive predictions with ENTPRISE-X having 56%, VEST-indel having 74% false predictions. Even at a cutoff of 0.9, ENTPRISE-X has 22% and VEST-indel has 36% false predictions for this particular data set. The high FDR for this set at the default cutoff value is mainly due to the overwhelmingly number of neutral variations (true positive/true neutral ~ 0.08). Even if all true positives are recalled, with 9% of neutral variations falsely classified as being disease-associated, the number of false predictions will be larger than that of correct positive predictions.

### Ten-fold cross-validation

In order to benchmark ENTPRISE-X for nonsense as well as frameshift mutations, we performed a ten-fold cross-validation of ENTPRISE-X using the complete training dataset. Proteins in the whole training set are clustered into clusters with a 35% sequence identity cutoff. We then randomly partitioned these clusters into ten sets. To test frameshift and nonsense mutations in each set, we use the mutations in the remaining nine sets for training. The test is done in three scenarios: (a) evaluating the test set on frameshift and nonsense mutations together; (b) evaluating the test set only on frameshift mutations, and (c) evaluating the test set only on nonsense mutations. The results are compiled in Table 3 which shows that performance of ENTPRISE-X for frameshift and nonsense variations are very similar. Overall, the false positive rate of ENTPRISE-X in cross-validation is 18%, much more than that of the VEST-indel test set of 9.1%. This could be due to some contamination of true positives in the neutral set. The false discovery rates are relatively smaller than those on the VEST-indel test set due to the fact that the relative abundance of true positives is larger in the Ten-fold cross validation set than in the VEST-indel test set. We also compare our method for this set to the DDIG-in method (VEST-indel has used the same dataset for training, thus not compared) and SIFT-indel [22]. The SIFT-indel result was obtained from the server at http://sift.jcvi.org/www/SIFT_chr_coords_indels_submit.html. Clearly, due to its larger false positive rate, DDIG-in has a much larger false discovery rate. SIFT-indel is better than DDIG-in and worse than ENTPRISE-X in terms of its MCC, F-score and false positive rate.

**Table 3:** Ten-fold cross-validation of ENTPRISE-X on the whole training set^a^.

Variation type	MCC	Sensitivity	Specificity	F-score	False positive rate	False discovery rate
**ENTPRISE-X**						
Frameshift & nonsense	0.655	0.851	0.815	0.871	18.5%	10.8%
Frameshift	0.616	0.871	0.815	0.909	18.5%	4.9%
Nonsense	0.619	0.806	0.815	0.789	18.5%	22.8%
**DDIG-in**						
Frameshift & nonsense	0.156	0.755	0.393	0.721	60.7%	31.0%
Frameshift	0.059	0.727	0.341	0.771	65.9%	17.8%
Nonsense	0.253	0.821	0.415	0.638	58.5%	47.9%
**SIFT-indel**						
Frameshift	0.201	0.837	0.367	0.842	63.3%	15.3%

^a^ To be fair to all methods, only the consensus mutations of the compared methods are evaluated.

### Large scale test on the ExAC set

We next performed large scale testing of ENTPRISE-X, VEST-indel and DDIG-in on the ExAC set. The results are compiled in Table 4. Again, results by VEST-indel and DDIG-in are obtained from their respective webserver. Consistent with the test on the VEST-indel test set, VEST-indel classifies around 10% more disease causing mutations than ENTPRISE-X for frameshift mutations. ENTPRISE-X classifies around 16% of both frameshift and nonsense mutations as disease causing. The fractions of disease causing mutations classified by DDIG-in are around 65% for both frameshift and nonsense mutations and are far too high. These rates are well correlated with the false positive rates in Table 2. The 16% rate of ENTPRISE-X is very close to the false positive rate for ten-fold cross-validation; indicating that the ExAC set is similar to the neutral part of the training set in terms of the probability of being contaminated with some true positives because ENTPRISE-X has roughly the same ~16% false positive rate for both sets.

**Table 4:** Comparison of the percentage of disease causing variations in the ExAC set^a^.

Method	Frameshift	Nonsense
	Evaluated #: 48123	Evaluated #: 40482
ENTPRISE-X	16.5%(0.73:6.0%)	15.7%(0.73:5.6%)
VEST-indel	26.2%(0.82:9.5%)	-
DDIG-in	64.4%(0.81:33.3%)	65.4%(0.81:35.1%)

^a^ To be fair to all methods, only the consensus mutations of three methods are reported. Numbers in parenthesis are cutoff:false positive rate using the cutoff that maximizes the MCC in Table 2.

### Statistical significance of ENTPRISE-X predictions

**Fig 3:**
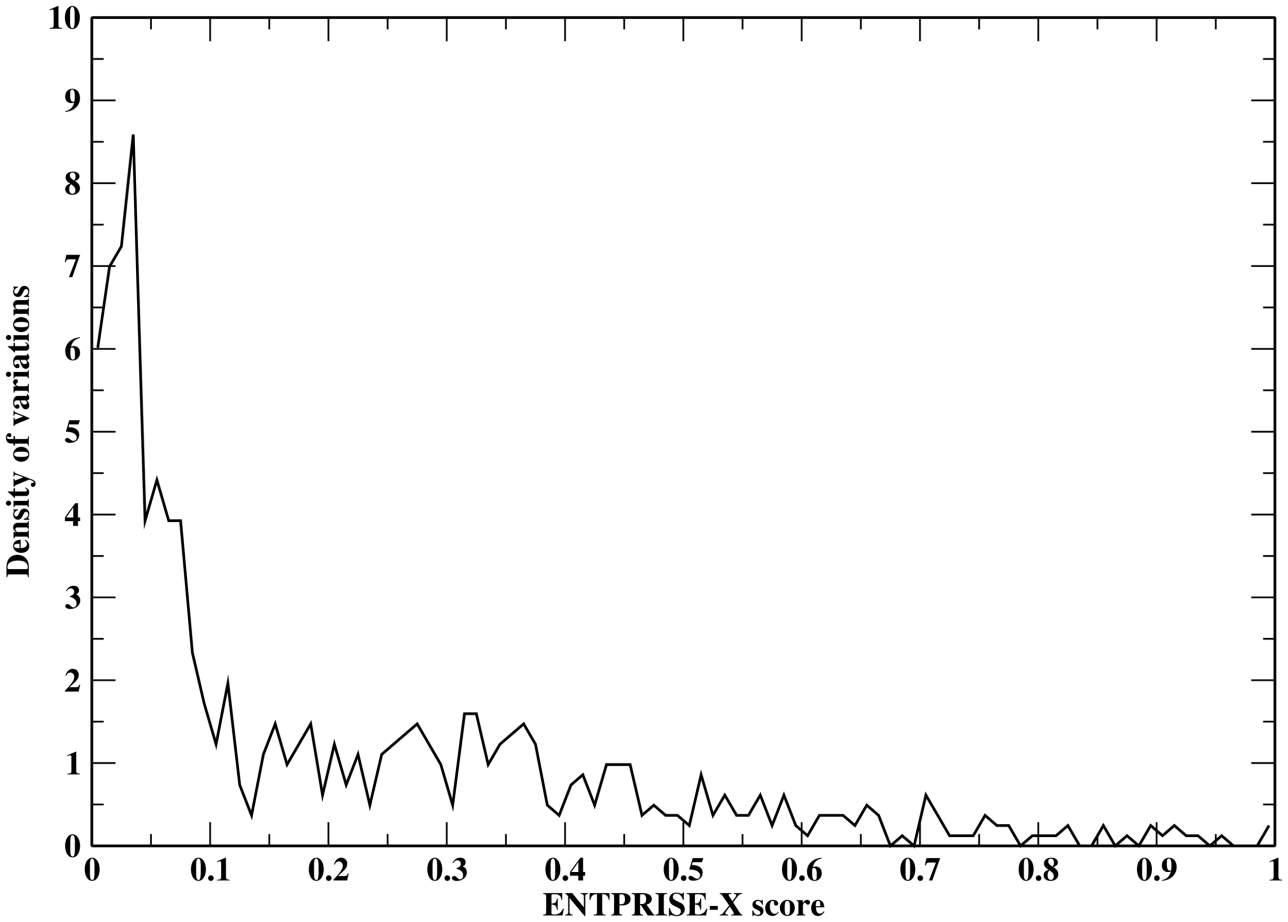
Distribution of ENTPRISE-X scores for the neutral variations in the VEST-indel test set. The area under the curve is normalized to one.

**Fig 4:**
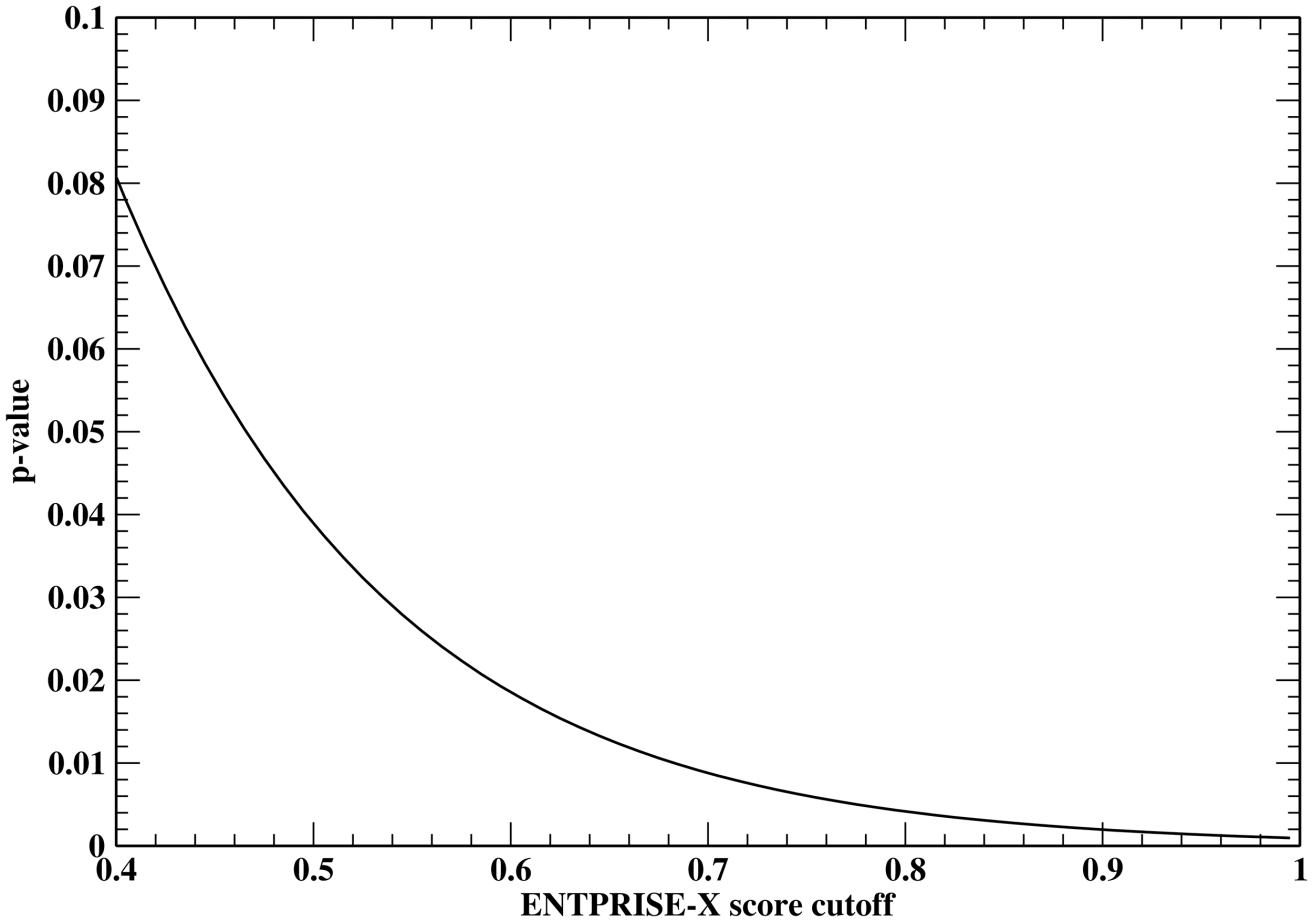
P-value derived from the VEST-indel test set by fitting to an extreme value distribution versus ENTPRISE-X score cutoffs.

In order to estimate the false positive rate at an arbitrary prediction score, a smooth fitting function of the false positive rate distribution is needed. This is equivalent to finding the p-value (the probability of falsely classifying a mutation being disease-associated when it is a true neutral at a given cutoff score) given an arbitrary prediction score. To derive a p-value measuring the statistical significance of ENTPRISE-X predictions, we utilize a total of 1,025 neutral frameshift mutations from the VEST-indel test set. The distribution of ENTPRISE-X scores is shown in Fig 3. The fitting function rapidly approaches zero at a score ~ 1. Based on the shape of the distribution in Fig 3, we tried fitting the curve with Normal, Weibull, Extreme value, Log-logistic and Burr distributions. We found that the extreme value distribution is the best two-parameter fit with mean μ = 0.0706 and variation σ = 0.1331 using least root mean squared deviation criterion. The p-value of score x by ENTPRISE-X can be obtained with $p(x)=1-exp[-\text{exp}\left(\frac{\mu -x}{\sigma }\right)]$. The default cutoff score 0.5 has a p-value of 0.039 that is smaller than the observed false positive rate 9% for the VEST-indel test set. This could be due to statistical fluctuations of the test data. Fig 4 shows the p-value against cutoffs. Similar fitting for VEST-indel score gives μ = 0.1371 and variation σ = 0.1583 that results in a p-value of 0.096 at the default cutoff 0.5.

**Fig 5:**
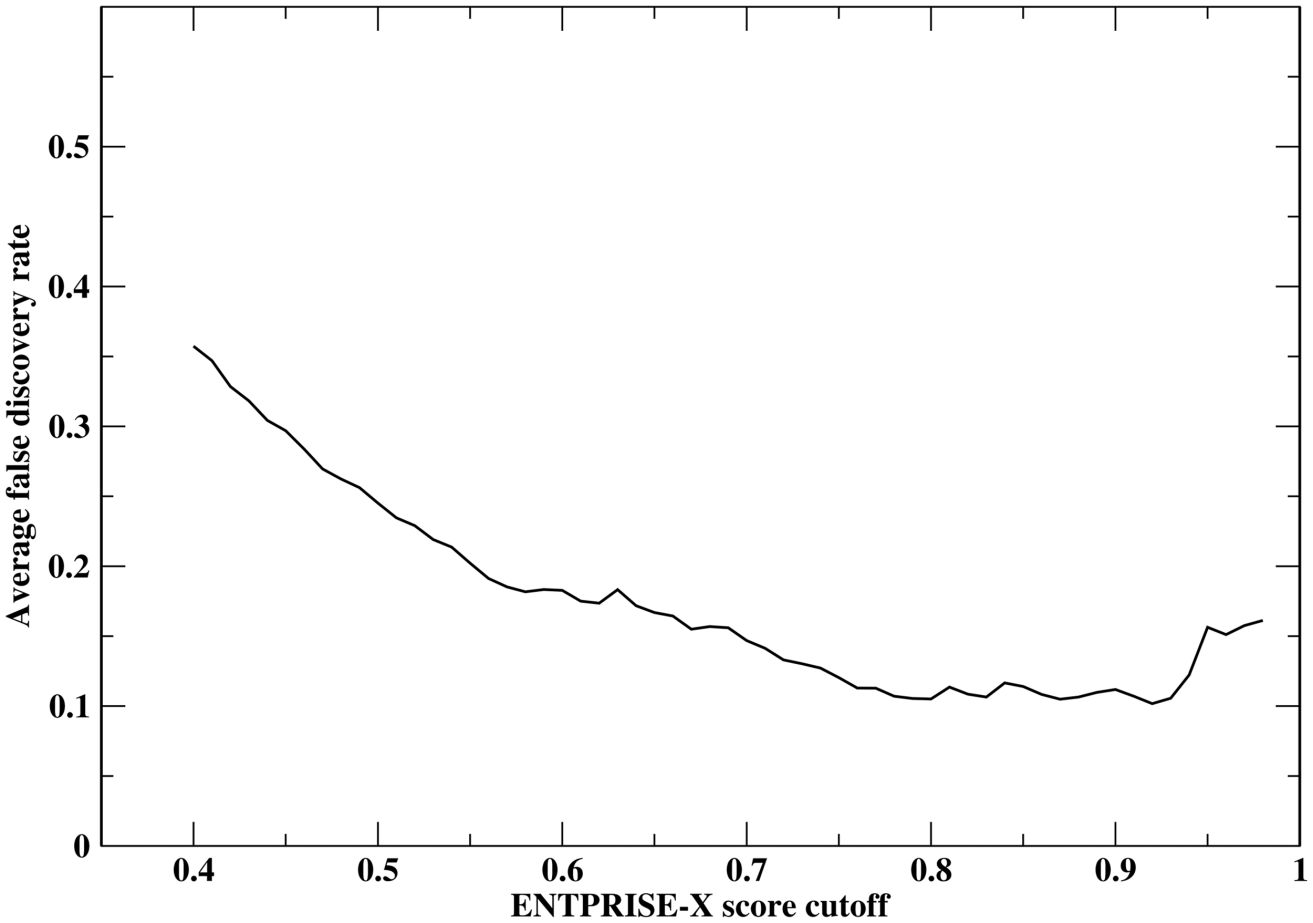
Dependence of average false discovery rate of ENTPRISE-X for 13 human exomes on score cutoffs.

Next, we apply the p-value that is equivalent to theoretical false positive rate at a given cutoff score to real exome data to estimate the false discovery rate of ENTPRISE-X prediction along with DDIG-in method. We obtained data from 13 individuals (private communication) and annotated their frameshift and nonsense variations using ENTPRISE-X. Table 5 summarizes the annotation results. The numbers of genes predicted to be disease-associated are around 30–70 when a default cutoff of 0.5 is used. Since the true neutral input variations are not known, here, we estimate the false predictions at given cutoff by *pN*_*total*_, where *p* is the p-value at the cutoff, *N*_*total*_ is the total number of genes evaluated as an approximation of the total number of neutral input genes. Then the false discovery rate can be estimated by *pN*_*total*_ / *N*_*pred*_, where *N*_*pred*_ is number of predicted genes to be disease-associated. The average false discovery rate over the 13 individuals is around 25% with the default cutoff 0.5. The dependence of the average false discovery rate for 13 human exomes on the cutoff is shown in Fig 5. It has a minimum at a cutoff score of 0.92 with a 10% false discovery rate and then it increases with increased cutoff. This is due to the observation that *N*_*pred*_ drops faster than the p-value does. Obviously, the FDRs of DDIG-in is too high to be practically useful as its FDR ranges from 0.55 to 0.88.

**Table 5:** Summary of patient annotations using ENTPRISE-X^a^.

patient	# of annotated variations	# of annotated genes	# of disease associated genes	false discovery rate
1	396/337	274/201	40/77	0.267/0.880
2	473/437	313/245	53/119	0.230/0.694
3	561/629	341/297	72/182	0.185/0.550
4	417/395	270/207	40/94	0.263/0.742
5	397/393	262/190	38/86	0.269/0.745
6	410/377	271/226	34/105	0.311/0.725
7	454/340	284/198	34/79	0.326/0.845
8	380/352	261/205	39/97	0.261/0.712
9	431/ 414	278/227	48/96	0.226/0.797
10	402/372	255/206	47/94	0.212/0.739
11	494/451	319/256	51/123	0.244/0.701
12	572/588	353/299	69/184	0.200/0.548
13	547/580	342/301	67/166	0.199/0.611

^a^ In each cell, first number is from ENTPRISE-X, second number is from DDIG-in method.

## Discussion

In this work, we have extended our machine learning approach ENTPRISE for predicting disease association of missense mutations to address frameshift and nonsense mutations in ENTPRISE-X. We show that ENTPRISE-X’s performance is superior to the state-of-the-art VEST-indel and DDIG-in methods. As with ENTPRISE, ENTPRISE-X has a much lower false positive rate than its peers while maintaining comparable sensitivity. In practice, for an entire exome, a large false positive rate will result in a method not useful because of the large number of mutations incorrectly identified as being disease-associated. The better performance of ENTPRISE-X demonstrates that selection of proper features and their integration yields better results. For example, the protein structure based local features help ENTPRISE-X to increase its sensitivity while maintaining specificity. As was shown for ENTPRISE [18], use of boosted tree regression gives superior performance compared to training using SVM based training. A natural extension of the current work is to place disease-causing genes in the content of a functional network to establish whether they would be useful disease-associated drug targets arising from missense mutations or if the protein is missing due to frameshift/nonsense mutations, what is the best downstream protein to target to treat the disease.

## Supporting information

S1 File All training and testing data sets. (ZIP)
